# A novel activating somatic mutation in *EPAS1*, coding for HIF-2α, in a patient with a paraganglioma and sickle cell disease

**DOI:** 10.20945/2359-4292-2026-0027

**Published:** 2026-04-01

**Authors:** Kalyan Mansukhbhai Shekhda, Rishi Iyer, Mercedes Robledo, Viktorija Nara, Tu Vinh Luong, Martyn Caplin, Ashley B Grossman

**Affiliations:** 1 Neuroendocrine Tumour Unit, ENETS Centre of Excellence, Royal Free Hospital NHS Foundation Trust, London, UK; 2 Hereditary Endocrine Cancer Group 204 EA, Human Cancer Genetics Programme, Spanish National Cancer Centre (CNIO), Madrid, Spain; Centro de Investigación Biomédica en Red de Enfermedades Raras (CIBERER), Madrid, Spain; 3 Department of Cellular Pathology, Royal Free Hospital NHS foundation trust, London, UK; 4 University College London School of Medicine, London, UK; 5 Green Templeton College, University of Oxford, Oxford, UK

**Keywords:** *EPAS1* gene, HIF-2α, sickle cell disease, paraganglioma, hypoxia inducible factor

## Abstract

Pheochromocytomasand paragangliomas (collectively referred as PPGLs) are highly heritable neoplasms arise from chromaffin cells of neural crest tissues; 40% of patients with PPGLs harbour germline pathogenic variants (PV), which up to 45% of patients exhibit somatic mutations in similar susceptibility genes. Endothelial PAS domain-containing protein-1 [also known as hypoxia inducible factor-2α, HIF-2α] is encoded by *EPAS1*, and along with other hypoxia-inducible factors (HIFs) acts as a key mediator in the cellular response to hypoxia. Gain-of-function mutations in *EPAS1* have been linked to the Pacak-Zhuang syndrome, congenital cyanotic heart disease and sickle cell anaemia. Hypoxia due to chronic anaemia and/or associated nephropathy in patients with sickle cell disease (SCD) may increase the expression of genes related to HIFs, thereby increasing susceptibility to the development of PPGLs. We describe a case of young female with a history of sickle cell anaemia and sickle cell nephropathy who was found to have a para-aortic mass. Histology confirmed the diagnosis of a paraganglioma. She did not exhibit somatic mutations of the common predisposition genes but demonstrated a likely pathogenic activating somatic *EPAS1* variant mutation. This case illustrates the predisposition of patients with SCD to PPGLs due to somatic *EPAS1* mutations, and should increase awareness of such tumours in these patients.

## INTRODUCTION

Pheochromocytomas and paragangliomas (PPGLs) are rare neuroendocrine tumours originating from chromaffin cells derived from the neural crest, and are notable for their high metastatic potential. Approximately 40% of PPGLs are associated with germline pathogenic variant (PVs) in one of more than 20 genes, including *VHL, RET, SDHx, MAX, FH, NF1* and *TMEM127* (^1^). More recently, somatic PVs in similar genes have been identified in 30-45% of PPGLs (^2^,^3^). The *EPAS-1* gene, which encodes endothelial PAS domain-containing protein-1 (also known Hypoxia Inducible Factor-2α (*HIF-2*α) (^4^), plays a critical role in oxygen sensing and tumorigenesis. Mosaic gain-of-function mutations in *EPAS1* have been described in the Pacak-Zhuang syndrome, a disorder characterised by congenital erythrocytosis, somatostatinomas and multifocal PPGLs, as well as retinal abnormalities (^5^). In addition, somatic *EPAS1* mutations have been reported in patients with sporadic PPGLs (^6^,^7^).

Sickle cell disease (SCD) is a chronic haemoglobinopathy associated with intermittent and sustained hypoxia, resulting from recurrent vaso-occlusion and anaemia. It has been proposed that chronic hypoxia in SCD may promote the development of PPGLs through the acquisition of pathogenic somatic *EPAS1* variants (^8^). We report a case of a somatic *EPAS1* mutation in a patient with a paraganglioma and sickle cell anaemia, supporting a potential link between chronic hypoxic states and tumorigenesis.

## CASE PRESENTATION

A 33-year-old Afro-Caribbean lady presented to the emergency department with a history of left flank pain. She had no history of episodic flushing, palpitations, sweating or headache. She had moved to the UK from Nigeria approximately one year prior to her presentation. On examination, her blood pressure was 120/77 mmHg, pulse rate of 80 beats per minute. She had a history of sickle cell anaemia since childhood, with frequent hospital admissions associated with painful vaso-occlusive crises as a child, with a brief pause in her 20s followed by further episodes of vaso-occlusive crises. She also had suffered with several episodes of an ‘acute chest syndrome’, a life-threatening complication of sickle cell disease characterised by fever, cough, chest pain and new lung infiltrates on a chest X-ray, requiring blood transfusions. Due to her chronic and active disease, she also required exchange transfusion prior to any surgical intervention. She was started on hydroxyurea in Nigeria, but she took this only inconsistently. She had no pregnancies in the past, no sickle cell retinopathy and no skin ulcers. Her urine protein/creatinine ratio was 385.7 mg/mmol (normal range: < 15 mg/mmol for non-pregnant adults). Ultrasound of her kidneys was unremarkable.

Her blood test results revealed a haemoglobin of 73 g/L (normal range for women: 115-165 g/L), MCV (mean corpuscular volume) 91.4 fL (80-100 fL), white cell count 11.4 x 10^9^/L (normal range: 4.00 to 11.00 x 10^9^/L), urea 9.1 mmol/L (normal range: 2.5 to 7.8 mmol/L), creatinine 109 mmol/L, with an eGFR of 58 ml/min (normal value: > 80 mL/min).

**Figure 1:**
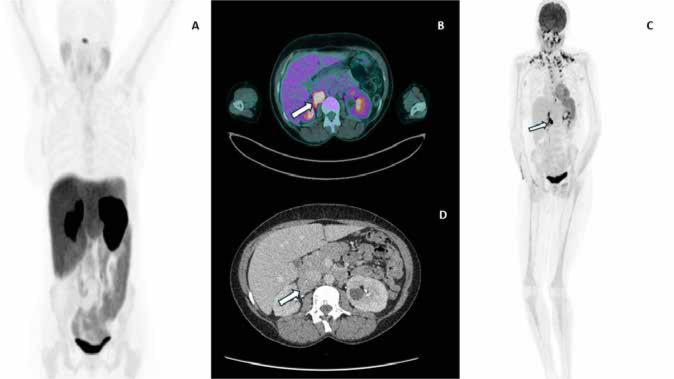
(**A**) ^68^Ga-DOTATATE scan shows no DOTATATE avid mass; (**B, C**) ^18^FDG-PET scan shows an intensely FDG-avid soft tissue mass on the right side (white arrow); **D:** CT abdomen and pelvis show a 26 x 34 mm mass adjacent to right renal vein, duodenum and inferior vena cava.

She was restarted on hydroxyurea 1 gm once a day with a view to prevent further vaso-occlusive crises, ampicillin for infection prophylaxis, and ramipril 1.25 mg for renal protection. However, her routine radiological investigations revealed a right-sided soft tissue mass posterior to the inferior vena cava (IVC). Functional imaging with Fluoro-18-deoxy-glucose Positron Emission Tomography (^18^FDG-PET) scanning and Gallium-68 1,4,7,10-tetraazacyclodo-decane-1,4,7,10-tetraacetic acid (DOTA)-octreotate (^68^Ga-DOTATATE) scanning revealed the mass to be intensely FDG-avid, but DOTATATE non-avid (**Figure 1**).

**Figure 2:**
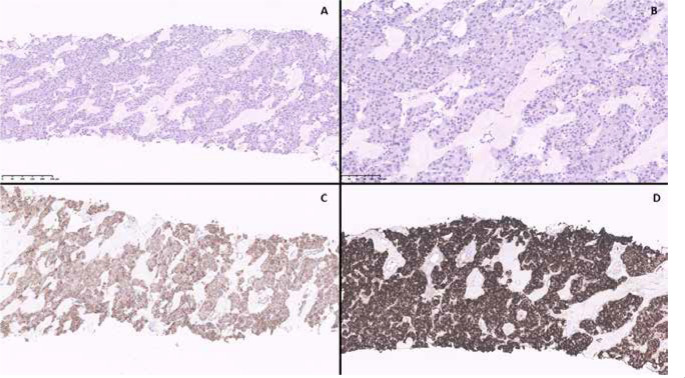
(**A**) H&E-stained section (x10) showing extensive infiltration by neoplastic cells. (**B**) H&E-stained section (x20) showing thick trabeculae of epithelioid cells divided by fibrous bands. (**C**) Immunohistochemistry (IHC) staining (x10) showing diffusely retained, cytoplasmic positivity for *SDHB.* (**D**) Immunohistochemistry (IHC) staining (x10) showing diffuse positivity for chromogranin A.

Histologically, the retrocaval soft tissue core biopsy revealed extensive infiltration of fibrous tissue by a neoplasm composed of confluent irregular trabecular structures. Tumour cells had abundant amphophilic cytoplasm and small to medium-sized, round or oval nuclei without visible nucleoli. Immunohistochemistry revealed the tumour cells to be strongly and diffusely positive for chromogranin, synaptophysin, S100, GATA3, and SDHB-positive. The epithelial marker MNF116 was negative. The Ki-67 proliferation index was < 1%. All immunohistochemical findings confirmed the diagnosis of a paraganglioma (**Figure 2**).

**Supplementary Figure 1:**
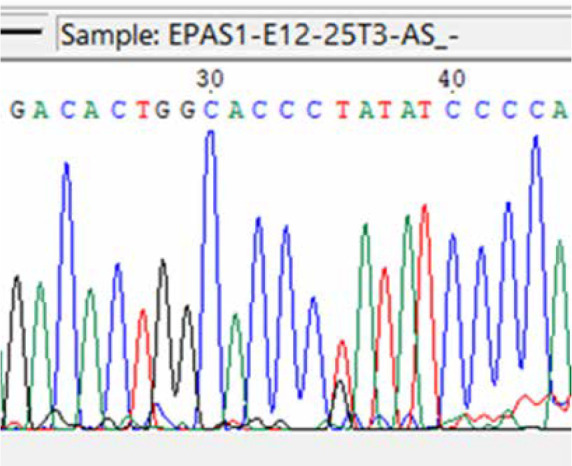
According to ClinGen/CGC/VICC guidelines, the variant was classified as likely oncogenic. Furthermore, previous functional studies have demonstrated that mutations affecting residues 530-532 impair prolyl hydroxylation of the HIF-2α protein, thereby disrupting its oxygen-dependent degradation. This evidence supports the oncogenic character of the variant.

Somatic mutation analysis identified a likely pathogenic variant in *EPAS1* (Exon 12, c.1594T>G, p.Tyr532Asp) with a variant allele frequency (VAF) of 39.9%, which was subsequently validated by Sanger sequencing (**Supplementary Figure 1**). According to ClinGen/CGC/VICC guidelines for somatic variant oncogenicity, the variant scores 6 points, and thus is considered probably oncogenic when the score is between 6 and 9 (^9^). Furthermore, previous functional studies have demonstrated that mutations affecting residues 530-532 impair prolyl hydroxylation of the HIF-2α protein, thereby disrupting its oxygen-dependent degradation. This evidence supported the pathogenic character of the variant.

She was referred for the surgical removal of this mass; however, due to her being asymptomatic, she declined the surgery and decided to be followed up and monitored for the mass in a surveillance clinic. Further diagnostic work up with plasma metanephrines and assessment of her genetic germline mutation studies was advised, but she opted not to pursue these further.

## DISCUSSION

Pheochromocytomas and paragangliomas (PPGLs) are rare neuroendocrine tumours of neural crest origin, arising from chromaffin tissues throughout the body. Their prevalence increases with age, with the highest incidence observed in individuals aged 60-79 years (^10^). Paragangliomas most often arise along the thoracic and abdominal sympathetic chains, with abdominal paragangliomas accounting for up to 50% of all cases (^11^). Rarely, they may be found in other locations such as the urinary bladder (^12^). Up to 25% of paragangliomas are functional, characterised by episodic secretion of catecholamines, including adrenaline (epinephrine), noradrenaline (norepinephrine) and dopamine.

This present case illustrates a clinically relevant association between sickle cell disease and *EPAS1*-mutated paragangliomas, supporting the emerging hypothesis that chronic hypoxia exerts selection pressure favouring gain-of-function mutations in the HIF-2α (*EPAS1*) pathway.

The cellular response to hypoxia is predominantly mediated by hypoxia-inducible factors (HIF*s*), transcriptional complexes which serve as master regulators of oxygen homeostasis (^13^). Endothelial PAS domain-containing protein 1 (*EPAS1*), also known as Hypoxia-Inducible Factor 2-alpha (*HIF-2*α), is an *HIF-*α subunit of the transcription complex HIF-2. The stability of HIF-α subunits is oxygen-dependent. At normal oxygen concentrations, prolyl hydroxylation of HIF-α at specific proline residues creates a binding site for Von-Hippel-Lindau (VHL) tumour suppressor protein, subjecting the subunit to proteasomal degradation (^8^,^14^). During hypoxic conditions, this prolyl hydroxylase activity is inhibited, allowing stable HIFα subunits to form HIF-dimer complexes (^8^,^14^). Failure of prolyl hydroxylation activity due to gain-of-function mutations at prolyl residues will result in accumulation of HIF, leading to persistent activation of hypoxia-responsive genes in normoxic conditions. HIF activity promotes angiogenesis and cell proliferation (^8^).

The somatic *EPAS1* variant identified in our patient (c.1594T>G, p.Tyr532Asp) affects position 532 on exon 12. This mutation has not, to our knowledge, been previously reported. Previously functional studies have shown that mutations in residues 530-532 disrupt prolyl hydroxylation of the HIF-2α protein (^10^). With this variant lying at the critical hydroxylation site, this likely leads to increased stabilisation of HIF-2α and HIF activity, and therefore the Tyr532Asp mutation is almost certainly pathogenic by the same mechanism. The 39.9% variant allele frequency in our case suggests significant clonal expansion, consistent with the driver role of *EPAS1* mutations in PPGL tumorigenesis.

Somatic *EPAS1* mutations exist in patients with isolated PPGL (^10^), but have also been identified in association with erythrocytosis (^10^). In the Pacak-Zhuang syndrome, somatic mosaicism of PVs in *EPAS1* appears to cause PPGLs alongside polycythaemia, with or without somatostatinomas (^5^). However, while this condition is rare, recent studies have established a compelling link between chronic hypoxic conditions and *EPAS1*-mutated PPGLs (^5^-^8^). The prevalence of PPGLs in patients with cyanotic congenital heart disease is up to four times higher than in the normal population (^15^). In patients with cyanotic congenital heart disease with PPGLs, Vaidya and cols. reported a strikingly high 80% *EPAS1* mutation prevalence among PGLs developing in these patients, with a 94% positive mutation rate in a subsequent Japanese series. These findings support the hypothesis that chronic hypoxaemia may foster a selective environment that promotes the clonal expansion of cells harbouring gain-of-function mutations in *EPAS1* (^16^,^17^). This principle may also apply in SCD: White and cols. identified four patients with SCD and PPGL, all of whom had an *EPAS1* mutation (^8^). In a more recent study involving 40 patients with *EPAS1*-mutated paragangliomas, haemoglobin disorders were identified in 59% of cases where electrophoresis data were available. Of these, 6 patients had SCD, 5 had sickle cell trait, and two were heterozygous for haemoglobin C disease (^18^). These findings further support the mechanistic hypothesis that sickle cell haemoglobinopathy carriers have an increased susceptibility to developing *EPAS1*-driven PPGLs, although the actual prevalence in SCD, a common disorder in many parts of the world, is unknown.

Clinically, these *EPAS-1*-mutated PPGLs in SCD/sickle cell carriers occur in younger patients with a female predominance (^8^). They are usually indolent, but the tumours do carry metastatic potential (^19^). Our patient’s presentation aligns closely with these established patterns. The patient was a young female aged 33 years, the tumour was in the retroperitoneum and demonstrated typical histological features including a low proliferative index (Ki-67 < 1%) and retained *SDHB* expression. Current published studies and cases of *EPAS-1* mutation-related PPGL are summarised in **Tables 1** and **2**. While surgical treatment would be treatment of choice in these cases, some patients might want to avoid major surgical intervention, as in our case. However, knowledge of this somatic mutation is useful insofar as should there be tumour expansion, medical therapy with the HIF-2α antagonist belzutifan might be considered as treatment (^20^).

**Table 1:** Published studies on somatic mutations in *EPAS1* gene in patients with PPGL

Year, country	Type of study	Number of patients with EPAS1 somatic variants and the disease associated with it	Description
USA, Brazil, Spain, 2018 (^16^)	RS	4 out of 5 patients with PPGL and CCHD had somatic mutation in *EPAS1* gene	5 patients (age: 13 - 54 years) had synchronous PPGL and CCHD 4 patients (80%) out of 5 had somatic *EPAS1* mutation
Japan, 2022 (^17^)	RS	7 patients with PPGL complicated by CCHD. A total of 16 PPGL samples were analysed for somatic and germline mutations for *EPAS1*	*EPAS1* somatic mutation was found in 15 out of 16 CCHD-PPGL samples (94%). Median age of 26 years (18-46 years) Median cumulative duration of cyanosis of 26 years (range, 12-46 years)
UK, 2023 (^8^)	RS	4 patients out of 128 patients with PPGL had simultaneous diagnosis of sickle cell anaemia and PPGL. They were included in the study for somatic *EPAS1* mutation	*EPAS1* somatic mutations were found in all four patients. 3 out of 4 patients were female (75%) All were homozygous for HbSS 2-4 unplanned admissions to hospital for SCD related complications
France, 2024 (^18^)	RS	40 patients with *EPAS1* related PPGL	4 patients had cyanotic congenital heart disease 13 out of 22 patients (59%) with haemoglobin electrophoresis results had haemoglobinopathies (6 SCD, 5 SCT, 2 heterozygous haemoglobin C disease)

EPAS-1: endothelial PAS domain contain protein-1; Endothelial RS: retrospective; PPGL: pheochromocytoma and paraganglioma; CCHD: cyanotic congenital heart disease.

**Table 2:** Published cases of pheochromocytoma or paraganglioma with haemoglobinopathies

Year, country	Age at diagnosis, sex	Diagnosis	Somatic mutation status *EPAS1*	Description
1990, United Kingdom (^21^)	32 Female	PCC and HbSC disease	n/d	History of intermittent headache, palpitations during pregnancy Right sided 4 cm PCC Patient underwent caesarean section at 35 weeks of pregnancy followed by right adrenalectomy
2002, United Kingdom (^22^)	22 Male	PCC and HbSS	n/d	Patient with HbSS, required admission for frequent crisis Developed resistant hypertension, renal failure CT left adrenal PCC Removed successfully
2003, Ireland (^23^)	33 Female	PGL and HbSS	n/d	36 weeks pregnancy Presented with headache, malaise and hypertension Surgical removal of PGL post-partum No lymph node involvement
2003, USA (^24^)	13	PCC and HbSS	n/d	n/d
2005, Germany (^25^)	27 Female	PCC and HbS-beta thalassaemia	n/d	Incidental right adrenal mass during pregnancy detected on US scan Asymptomatic pregnancy with uneventful delivery Right adrenalectomy post-partum
2014, Sweden (^26^)	43 Female	PCC	Somatic *EPAS1* variant c.1589C>A. Not present in germline DNA	n/d
2016, USA (^27^)	n/d	Extra-adrenal paraganglioma	n/d	Metastatic PGL
2019, Nigeria (^28^)	34 Female	PGL and HbSS	n/d	Patient presented with generalised abdominal pain and distension Left retroperitoneal mass with extensive mesenteric and omental LN involvement Laparotomy and resection Died of multi-organ failure
2019, USA (^29^)	46 Male	PGL	n/d	Abdominal pain, refractory hypertension Surgical resection
Our case, UK	33 Female	PGL with sickle cell nephropathy	Somatic *EPAS1* mutation variant c.1594T>G, p. Tyr532Asp	Flank pain otherwise asymptomatic FDG avid and DOTATATE non-avid right infra-renal mass, no metastatic disease elsewhere Histology confirmed paraganglioma with Ki67 < 1%

PCC: pheochromocytoma; HbSC: haemoglobin SC disease; HbSS; sickle cell anaemia; PGL: paraganglioma.

Limitations: Genetic data on possible germline mutations could have provided additional insights and enhanced the diagnostic accuracy. However, this was not performed due to patient preference.

In conclusion, this case contributed to the growing body of evidence linking EPAS1 PV/PPV and PPGLs in SCD by identifying a novel somatic pathogenic variant (p.Tyr532Asp). It expands the known spectrum of PVs associated with this clinically relevant context. Currently, routine molecular evaluation in PPGLs primarily includes germline testing. However, this case underscores the importance of incorporating somatic sequencing, particularly in patients with SCD and PPGL, to guide personalised management and improve diagnostic precision.

## Data Availability

the data sets analysed in this study are included in the manuscript.

## References

[1] Buffet A, Burnichon N, Favier J, Gimenez-Roqueplo AP. (2020). An overview of 20 years of genetic studies in pheochromocytoma
and paraganglioma. Best Pract Res Clin Endocrinol Metab.

[2] Fishbein L, Leshchiner I, Walter V, Danilova L, Robertson AG, Johnson AR (2017). Comprehensive Molecular Characterization of Pheochromocytoma and
Paraganglioma. Cancer Cell.

[3] Nölting S, Bechmann N, Taieb D, Beuschlein F, Fassnacht M, Kroiss M (2022). Personalized Management of Pheochromocytoma and
Paraganglioma. Endocr Rev.

[4] Kristan A, Debeljak N, Kunej T. (2021). Integration and Visualization of Regulatory Elements and
Variations of the EPAS1 Gene in Human. Genes (Basel).

[5] Zhuang Z, Yang C, Lorenzo F, Merino M, Fojo T, Kebebew E (2012). Somatic HIF2A gain-of-function mutations in paraganglioma with
polycythemia. N Engl J Med.

[6] Comino-Méndez I, de Cubas AA, Bernal C, Álvarez-Escolá C, Sánchez-Malo C, Ramírez-Tortosa CL (2013). Tumoral EPAS1 (HIF2A) mutations explain sporadic pheochromocytoma
and paraganglioma in the absence of erythrocytosis. Hum Mol Genet.

[7] Welander J, Andreasson A, Brauckhoff M, Bäckdahl M, Larsson C, Gimm O (2014). Frequent EPAS1/HIF2α exons 9 and 12 mutations in
non-familial pheochromocytoma. Endocr Relat Cancer.

[8] White G, Nonaka D, Chung TT, Oakey RJ, Izatt L. (2023). Somatic EPAS1 Variants in Pheochromocytoma and Paraganglioma in
Patients With Sickle Cell Disease. J Clin Endocrinol Metab.

[9] Horak P, Griffith M, Danos AM, Pitel BA, Madhavan S, Liu X (2022). Standards for the classification of pathogenicity of somatic
variants in cancer (oncogenicity): Joint recommendations of Clinical Genome
Resource (ClinGen), Cancer Genomics Consortium (CGC), and Variant
Interpretation for Cancer Consortium (VICC). Genet Med.

[10] Leung AA, Pasieka JL, Hyrcza MD, Pacaud D, Dong Y, Boyd JM (2021). Epidemiology of pheochromocytoma and paraganglioma:
population-based cohort study. Eur J Endocrinol.

[11] Li P, Zhao D. (2016). A rare case of retroperitoneal paraganglioma - case report and
literature review. Transl Gastroenterol Hepatol.

[12] Shekhda KM, Palan JM, Albor CB, Wan S, Chung TT. (2025). A rare case of bladder paraganglioma treated successfully with
robotic partial cystectomy. Endocr Oncol.

[13] Semenza GL. (2014). Oxygen sensing, hypoxia-inducible factors, and disease
pathophysiology. Annu Rev Pathol.

[14] Kaelin WG, Ratcliffe PJ. (2008). Oxygen Sensing by Metazoans: The Central Role of the HIF
Hydroxylase Pathway. Mol Cell.

[15] Agarwal S, Jindal I, Balazs A, Paul D. (2019). Catecholamine-Secreting Tumors in Pediatric Patients With
Cyanotic Congenital Heart Disease. J Endocr Soc.

[16] Vaidya A, Flores SK, Cheng ZM, Nicolas M, Deng Y, Opotowsky AR (2018). EPAS1 Mutations and Paragangliomas in Cyanotic Congenital Heart
Disease. N Engl J Med.

[17] Ogasawara T, Fujii Y, Kakiuchi N, Shiozawa Y, Sakamoto R, Ogawa Y (2022). Genetic Analysis of Pheochromocytoma and Paraganglioma
Complicating Cyanotic Congenital Heart Disease. J Clin Endocrinol Metab.

[18] Mancini M, Buffet A, Porte B, Amar L, Lussey-Lepoutre C, Crinière L (2024). EPAS1-mutated paragangliomas associated with haemoglobin
disorders. Br J Haematol.

[19] Alzahrani AS, Alswailem M, Buffet A, Alghamdi B, Alobaid L, Alsagheir O (2024). EPAS1-related pheochromocytoma/paraganglioma. Endocr Relat Cancer.

[20] Kamihara J, Hamilton KV, Pollard JA, Clinton CM, Madden JA, Lin J (2021). Belzutifan, a Potent HIF2α Inhibitor, in the Pacak-Zhuang
Syndrome. N Engl J Med.

[21] Pattison J, Harrop-Griffiths AW, Whitlock JE, Roberts JC. (1990). Caesarean section in a patient with haemoglobin SC disease and a
phaeochromocytoma. Anaesthesia.

[22] Myers B, Donohue SM. (2002). A case of sickle-cell erythrocytosis occurring following renal
transplantation. Clin Lab Haematol.

[23] Donnelly JC, Cooley SM, O’Connell MP, Murphy JF, Keane DP. (2003). Pheochromocytoma, sickle cell disease and pregnancy: a case
report. J Matern Fetal Neonatal Med.

[24] Schultz WH, Ware RE. (2003). Malignancy in patients with sickle cell disease. Am J Hematol.

[25] Müssig K, Horger M, Häring HU, Gallwitz B. (2005). Pheochromocytoma in a patient with
sickle-beta-thalassemia. J Matern Fetal Neonatal Med.

[26] Welander J, Andreasson A, Brauckhoff M, Bäckdahl M, Larsson C, Gimm O (2014). Frequent EPAS1/HIF2α exons 9 and 12 mutations in
non-familial pheochromocytoma. Endocr Relat Cancer.

[27] Thomas T, Thomas D, French K, Blinder MA. (2016). Malignancy in Patients with Sickle Cell Disease: A Single Center
Observational Study. Blood.

[28] Egigba O, Osime C, Ekanem V, Jibril P. (2019). Two nights in one day: A case report of paraganglioma in sickle
cell disease and a review of the literature. Port Harcourt Med J.

[29] Fancher C, Bridges L, Conforti A. A (2019). Rare Case of Malignant Peri-Aortic Paraganglioma. Am Surg.

